# Unraveling sulfur chemistry in interstellar carbon oxide ices

**DOI:** 10.1038/s41467-022-34949-4

**Published:** 2022-11-22

**Authors:** Xiaolong Li, Bo Lu, Lina Wang, Junfei Xue, Bifeng Zhu, Tarek Trabelsi, Joseph S. Francisco, Xiaoqing Zeng

**Affiliations:** 1 Department of Chemistry, Shanghai Key Laboratory of Molecular Catalysts and Innovative Materials, Fudan University, 200433 Shanghai, China; 2 Department of Earth and Environment Science, University of Pennsylvania, Philadelphia, PA 19104‑6243 USA

**Keywords:** Astronomy and planetary science, Planetary science

## Abstract

Formyl radical (HCO•) and hydroxycarbonyl radical (HOCO•) are versatile building blocks in the formation of biorelevant complex organic molecules (COMs) in interstellar medium. Understanding the chemical pathways for the formation of HCO• and HOCO• starting with primordial substances (e.g., CO and CO_2_) is of vital importance in building the complex network of prebiotic chemistry. Here, we report the efficient formation of HCO• and HOCO• in the photochemistry of hydroxidooxidosulfur radical (HOSO•)–a key intermediate in SO_2_ photochemistry–in interstellar analogous ices of CO and CO_2_ at 16 K through hydrogen atom transfer (HAT) reactions. Specifically, 266 nm laser photolysis of HOSO• embedded in solid CO ice yields the elusive hydrogen‑bonded complexes HCO•···SO_2_ and HOCO•···SO, and the latter undergoes subsequent HAT to furnish CO_2_···HOS• under the irradiation conditions. Similar photo-induced HAT of HOSO• in solid CO_2_ ice leads to the formation of HOCO•···SO_2_. The HAT reactions of HOSO• in astronomical CO and CO_2_ ices by forming reactive acyl radicals may contribute to understanding the interplay between the sulfur and carbon ice-grain chemistry in cold molecular clouds and also in the planetary atmospheric chemistry.

## Abstract (web summary)

The authors report photolytic H atom transfer reactions of HOSO• in astronomical CO & CO_2_ ices, forming reactive acyl radicals & molecular complexes with SO & SO_2_. Connecting the photochemistry of S oxides & C oxides in cold molecular clouds in the interstellar medium.

## Introduction

Sulfur is the tenth most abundant element in the universe, and sulfur chemistry plays vital importance not only in the biological systems and atmosphere on the Earth but also in interstellar medium (ISM). According to astronomical observations, the two sulfur oxides SO_2_ and SO have been found to be abundant in molecular clouds^1–4^, which are mainly formed through condensation of gas-phase molecules at the surface of dust grains (mostly amorphous silicates) with an onion-like structure at the temperatures of about 10–20 K^5,6^. The inner layer of the grains mainly consists of hydrogenated ice (H_2_O) with low concentrations of other H-containing species such as CH_3_OH, NH_3_, and CH_4_. The outer layer is made up of dehydrogenated ices with dominant compositions of CO, CO_2_, N_2_, O_2_, and SO_2_, and low concentrations of H_2_O may also be present in the outer layer of the icy mantle. The icy mantle at the surface of cosmic dust grains are the most important carriers of prebiotic molecules, and the composition of the mantles are largely affected by the exchanges between solid ice and gas-phase and also the photochemistry promoted by cosmic irradiations, including UV and X-ray photons from nearby stars. Therefore, the study about the chemical composition of the icy grains and the complex reaction networks is crucial for understanding the evolution of the molecular clouds^7–9^.

Carbon monoxide (CO) is the most abundant composition in the outer layer of icy grains in interstellar medium, and CO-abundant ices have also been found at the surface of many cold interstellar bodies, including comets, icy moons, and planets in the outer solar system. Therefore, the chemistry of CO through successive hydrogen atom addition reactions in the CO-rich outer layer of the interstellar icy grains may play a key role for the formation of complex organic molecules (COMs), which are probably building blocks for the origin of life^10–12^. Recently, it has been shown that simple radicals bearing elements C, N, O, P, or S play vital importance in prebiotic synthesis^13^. Reaction networks of these radicals in interstellar ice grains and the corresponding geochemical scenarios may help in unveiling the chemical evolution and origins of life. For instance, formyl radical (HCO•) and hydroxycarbonyl radical (HOCO•) are important intermediates in atmospheric and combustion chemistry^14^, and they are also versatile building blocks in the interstellar formation of biorelevant COMs such as formic acid (HC(O)OH)^15^, glyoxylic acid (HC(O)C(O)OH)^16^, and pyruvic acid (CH_3_C(O)C(O)OH)^17^ in low temperature (<30 K) interstellar CO and CO_2_ ices doped with H-containing species CH_4_ and H_2_O through barrierless radical-radical association reactions, in which the reactive acyl radicals can be generated through the hydrogenation of carbon oxides with the H-containing molecules embedded in the same ice layer at cosmic radiations^18,19^. As a simple organic species, HCO• has been observed in many interstellar clouds such as DR 21, Sgr B2, and NGC 2024^20,21^, and its radical recombination reaction with •CH_2_OH in producing COMs during the phase transition of interstellar CO ices doped with CH_3_OH and H_2_O at a typical dense cloud temperature of about 10 K has been recently disclosed^22–24^. The cationic form of HOCO• has been also identified in star-forming regions such as SgrB2(OH) and low‑mass protostar IRAS 04368 + 2557 in L1527^25,26^.

In sharp contrast to the extensively explored mechanisms for the formation of COMs through the photoreactions of H-containing species (e.g., CH_3_OH, NH_3_, and CH_4_) via the intermediacy of organic radicals such as HCO•, HOCO•, and CH_3_O• in interstellar icy grain mantles, the ice-grain photochemistry of the typical dehydrogenated molecules such as SO, SO_2_, and the derived sulfur-containing radicals HOS• and HOSO• in astronomical CO and CO_2_ ices remains barely investigated. On the other hand, the photochemistry of SO and SO_2_ also has great impact on the sulfur cycle in planetary atmospheres due to the formation and evolution of hazes and clouds in the upper atmospheres of Solar system planets such as Earth^27^, Venus^28^, Jupiter^29^, and the moon Io^30^. Among these sulfur oxides, SO_2_ is one of the most common pollutant in the Earth’s atmosphere. Therefore, the SO_2_ photochemistry has been the focus of enormous attention due to the important role in sulfur cycle by forming sulfuric acid and sulfate aerosols. According to the recent modeling studies^31,32^, the tropospheric photochemistry of SO_2_ in the presence of water proceeds mainly through the formation of HOSO• and hydroxyl radical (•OH) after absorption of light in the near UV‒vis range (250–340 nm). In contrast, the UV photolysis (190–220 nm) of SO_2_ in the gas phase leads to fragmentation by yielding SO^33^, which is an interstellar species that has been detected in the atmospheres of Venus^34^ and Io^35^. Chemically, SO is more reactive and it dimerizes easily to yield elemental sulfur and SO_2_ via the intermediacy of OSSO, and the dimer has been recognized as a candidate species that contributes to the mysterious near‑UV absorption (320–400 nm) in the yellowish atmosphere of Venus^36–38^.

**Fig. 1:**
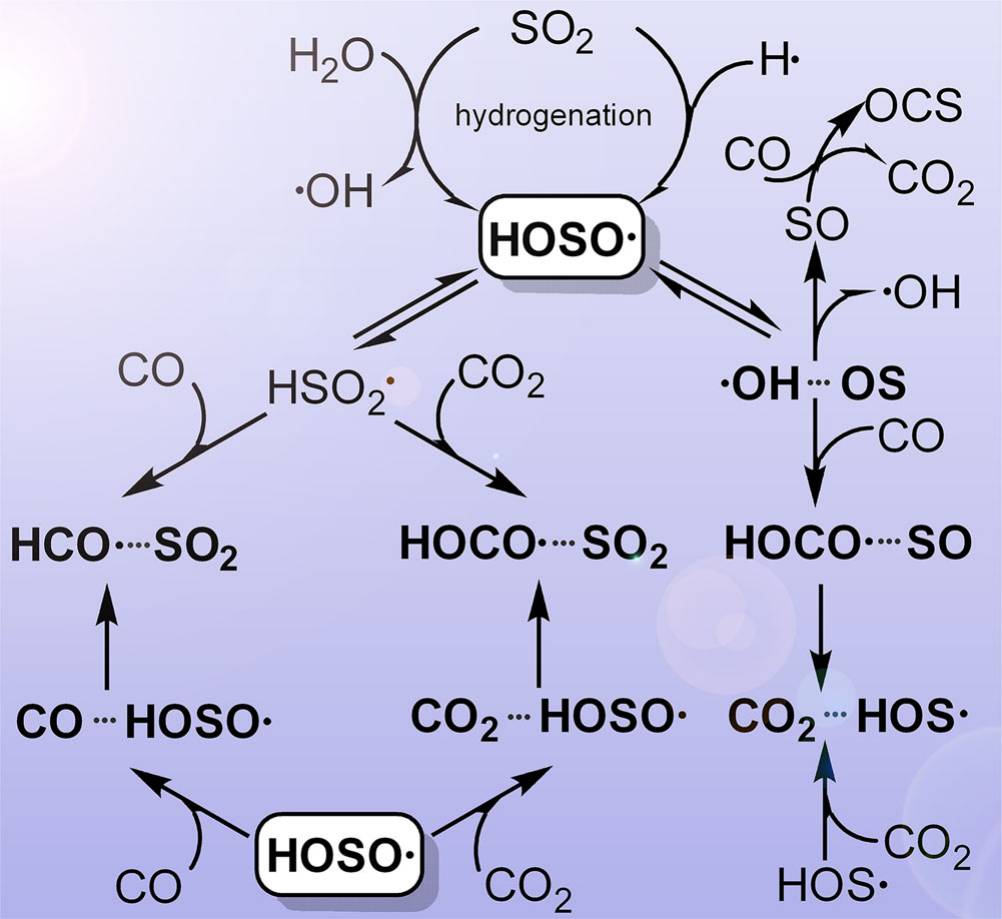
Photochemistry of HOSO• in solid CO and CO_2_ ices. Proposed pathways for the photo-induced decomposition, isomerization, and hydrogen atom transfer reactions of HOSO• in solid CO and CO_2_ at 16 K.

Herein, we report an experimental study on the photochemistry of the astrochemically relevant sulfur-containing species HOSO•, HOS•, SO_2_, SO, and OSSO in solid CO and CO_2_ ices at 16 K (Fig. 1). In addition to the molecular complexes formed between HOSO• and the carbon oxides, the photo-induced hydrogen atom transfer (HAT) to form new complexes consisting of acyl radicals (HCO• and HOCO•) and sulfur oxides (SO_2_ and SO) has been observed. It is noteworthy that weakly bonded molecular complexes consisting of interstellar species have been considered as potent contributors to the rich chemistry in low-temperature giant molecular clouds^39^.

## Results and Discussion

### Isolation of HOSO• in CO and CO_2_ ices

**Fig. 2:**
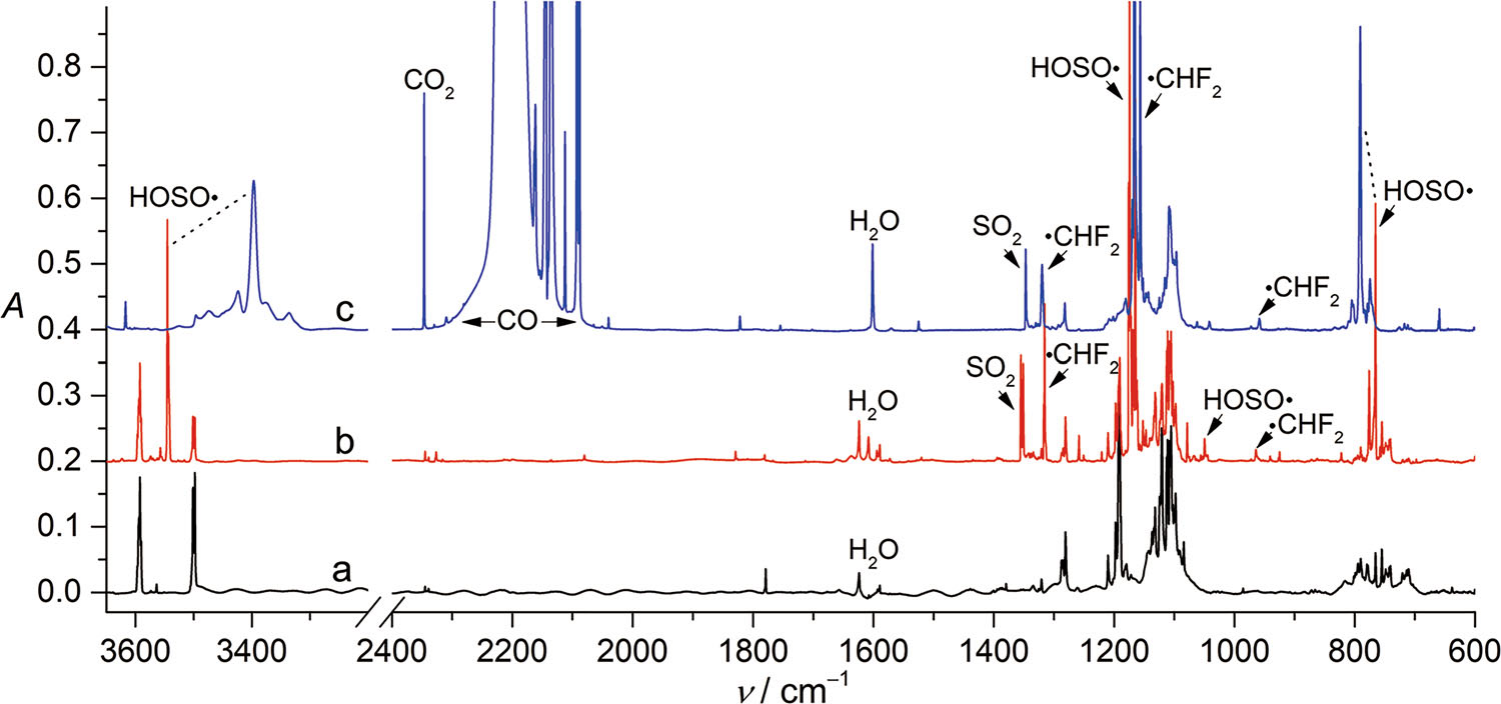
Infrared (IR) spectrum of CHF_2_S(O)OH and HOSO• in matrixes. **a** IR spectrum of CHF_2_S(O)OH in Ar-matrix at 10 K. **b** IR spectrum for the high‑vacuum flash pyrolysis (HVFP, ca. 700 °C) products of CHF_2_S(O)OH in Ar-matrix at 10 K. **c** IR spectrum for the HVFP (ca. 700 °C) products of CHF_2_S(O)OH in CO-matrix at 16 K.

Thanks to the strong “cage effect” of the solid host matrix materials (e.g., Ne and Ar) at low temperatures (<30 K), the matrix isolation technique has been broadly applied in trapping highly unstable intermediates, including weakly bonded molecule‑radical complexes such as •OH···CO^18^, •C_6_H_5_···H_2_O^40^, •OC_6_H_5_···H_2_O^41^, •OH···H_2_O^42^, and •NH_2_···H_2_O^43^. Recently, it has been shown that HOSO• can be efficiently generated in the gas phase through high‑vacuum flash pyrolysis (HVFP, ca. 700 °C) of CHF_2_S(O)OH (Fig. 2a), and photolysis of HOSO• (Fig. 2b) in solid Ar‑matrix at 10 K yields isomeric HSO_2_•, fragments SO_2_/H• together with the caged radical complex •OH···OS^44^. The absence of free fragments •OH and OS in the matrix suggests that they can hardly escape from the rigid matrix cages. When the pyrolytic generation of HOSO• was performed in the presence of CO by using a 1:20:1000 mixture of CHF_2_S(O)OH/CO/Ar, the IR spectrum of the isolated species (Supplementary Fig. 1) shows the appearance of new IR bands in the range of 3500–3350 cm^−1^ for O–H stretching vibrations (*ν*(OH)), implying complex formation between HOSO• and CO.

When using neat CO as the matrix host material, the IR spectrum of the isolated pyrolysis products at 16 K (Fig. 2c) clearly shows the absence of all the IR bands for free HOSO• (Fig. 2b), while the bands for the complex CO···HOSO• become dominant. The *ν*(OH) mode in HOSO• shifts from 3545.3 cm^−1^ in Ar‑matrix to 3396.8 cm^−1^ in CO‑matrix, corresponding to a red‑shift (Δ*ν*) of –148.5 cm^−1^. It is comparable with the shift of the *ν*(OH) mode in CO‑matrix isolated HOCO• (Δ*ν* = –146.9 cm^−1^) comparing to its IR spectrum in Ar‑matrix^45^. The assignment of CO···HOSO• is supported by the good agreement with the theoretically calculated shift of –140 cm^−1^ at the B3LYP‑GD3(BJ)/def2‑TZVP level (Table 1). In line with a stable hydrogen-bonded structure through OC···H bond in the complex, the stretching mode for the terminal S = O moiety (*ν*(S = O)) is less perturbed than the S–O stretching mode (*ν*(S–O)) as indicated by the shifts of –3.6 and +25.3 cm^−1^, respectively. In contrast, the weak deformation mode *δ*(SOH) exhibits a large blue‑shift of +44.2 cm^−1^, which is in agreement with the calculated shift of +53 cm^−1^ at the CCSD(T)/aug‑cc‑pV(T + D)Z level. Note there are several weak satellite bands around the main peaks for the fundamental modes of the complex, they probably arise from the less stable matrix sites or the less abundant complexes of HOSO• consisting two or more CO molecules.

**Table 1:** Calculated and observed Infrared (IR) spectra of HOSO• in different matrixes

HOSO•				CO···HOSO•			CO_2_···HOSO•		
Mode	Obs.^[a]^	B3LYP^[b]^	CCSD(T)^[c]^	Obs.^[d]^	B3LYP^[b]^	CCSD(T)^[c]^	Obs.^[e]^	B3LYP^[b]^	CCSD(T)^[c]^
*ν*(O–H)	3545.3	3717 (91)	3736	3396.8	3577 (469)	3640	3478.7	3633 (311)	3663
*ν*(S = O)	1168.2	1178 (104)	1183	1164.6	1182 (103)	1192	1163.8	1176 (136)	1186
*δ*(SOH)	1049.7	1062 (16)	1081	1093.9	1125 (8)	1134	n.o.	1108 (6)	1121
*ν*(S–O)	765.6	766 (188)	781	790.9	788 (173)	797	788.0	790 (167)	802

[a] Observed IR frequencies in Ar-matrix. [b] Calculated harmonic IR frequencies and intensities (km mol^−1^, in parentheses) at the B3LYP‑GD3(BJ)/def2-TZVP level of theory. [c] Calculated harmonic IR frequencies at the CCSD(T)/aug‑cc‑pV(T + d)Z level of theory. [d] Observed IR frequencies in CO-matrix. [e] Observed IR frequencies in CO_2_-doped Ar-matrix.

By analogy, deposition of the pyrolysis products of a 1:50:1000 mixture of CHF_2_S(O)OH/CO_2_/Ar at 16 K leads to the formation of the complex CO_2_···HOSO• (Supplementary Fig. 2). Consistent with the CCSD(T) calculated red-shift of –73 cm^−1^ (B3LYP: –84 cm^−1^) for the *ν*(OH) mode in the complex, the band for HOSO• shifts from 3545.3 cm^−1^ in Ar‑matrix to 3478.7 cm^−1^ in CO_2_‑matrix (Table 1), corresponding to a shift of –66.6 cm^−1^. Concomitantly, the *ν*(S–O) mode undergoes a blue-shift by +22.4 cm^−1^ (CCSD(T): + 21 cm^−1^; B3LYP: + 24 cm^−1^).

**Fig. 3:**
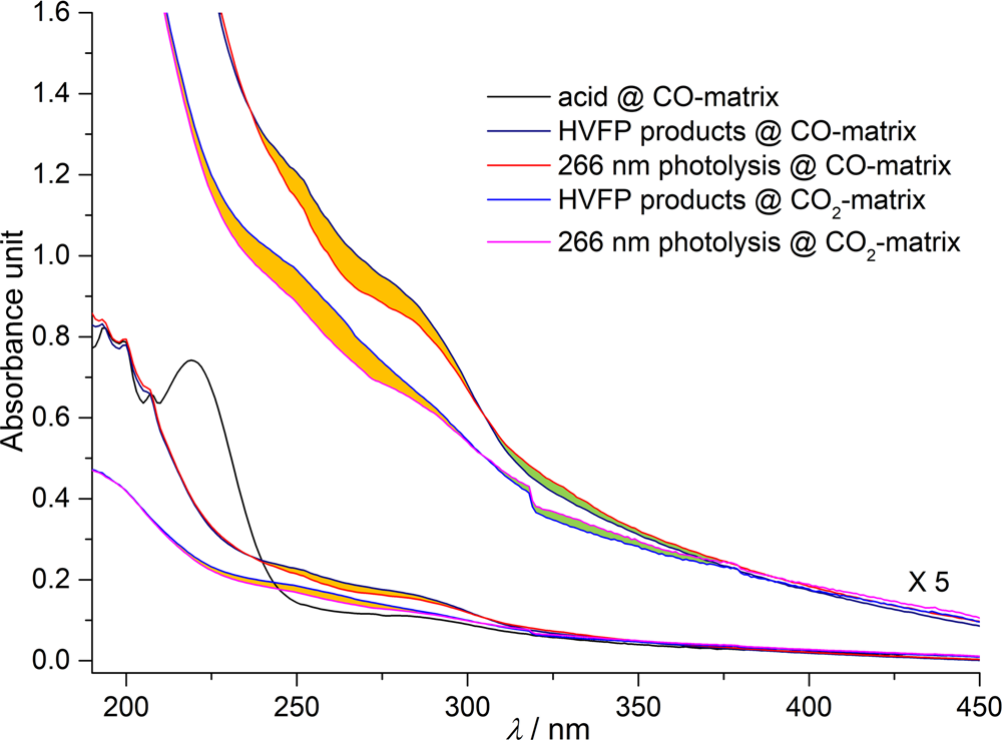
Ultraviolet-visible (UV‒vis) spectra of the acid precursor CHF_2_S(O)OH and its high-vacuum flash pyrolysis (HVFP) products isolated in solid CO and CO_2_ ices at 16 K. The absorptions for HOSO• and its photolytic reaction products in the matrixes are rendered with orange and green colors, respectively.

Weak hydrogen‑bonding interactions of HOSO• with carbon oxides also affect its UV-vis absorption. Recently, a broad absorption centered at 270 nm (*λ*_max_) has been observed for HOSO• in Ar-matrix, corresponding to the transition from the ground state (X^2^A) to the C^2^A/D^2^A excited states^46^. As shown in Fig. 3, the major absorption of HOSO• in CO‑matrix appears in the range of 350–240 nm as a weak band, and its assignment is ascertained with the photochemistry that HOSO• can be efficiently depleted by UV‑light irradiation at 266 nm^47^. This characteristic absorption for HOSO• is also observable in CO_2_‑matrix. In sharp contrast to the appearance of the absorption of HSO_2_• in the range of 320 − 500 nm after the irradiation (266 nm) of HOSO• in Ar‑matrix, same photolysis in CO‑ and CO_2_‑matrixes results in the occurrence of weaker absorptions in the range of 300 − 400 nm, implying the formation of new species arising from the photoreactions of HOSO• with the carbon oxides.

### Photochemistry of HOSO• in CO and CO_2_ ices

**Fig. 4:**
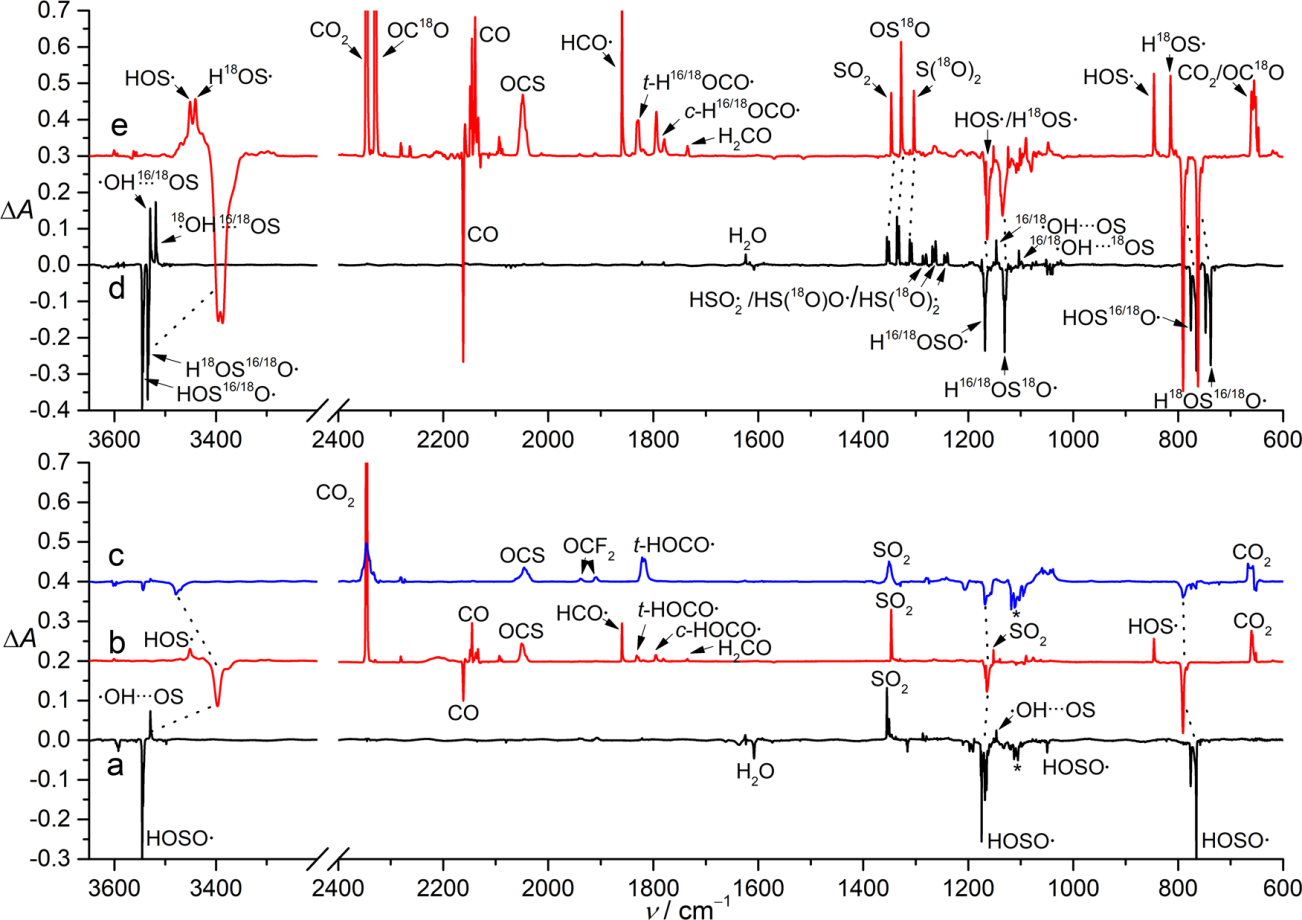
Reactions of HOSO• in matrixes upon irradiation. **a** Infrared (IR) difference spectrum reflecting the change of the Ar‑matrix isolated high‑vacuum flash pyrolysis (HVFP) products of CHF_2_S(O)OH upon irradiation at 266 nm (80 min, 10 K). **b** IR difference spectrum reflecting the change of the CO‑matrix isolated HVFP products of CHF_2_S(O)OH upon irradiation at 266 nm (7 min, 16 K). **c** IR difference spectrum reflecting the change of the CO_2_‑doped Ar-matrix (50: 1000) isolated HVFP products of CHF_2_S(O)OH upon irradiation at 266 nm (30 min, 16 K). **d** IR difference spectrum reflecting the change of the Ar‑matrix isolated HVFP products of ^18^O‑labeled CHF_2_S(O)OH upon irradiation at 266 nm (32 min, 10 K). **e** IR difference spectrum reflecting the change of the CO‑matrix isolated HVFP products of ^18^O‑labeled CHF_2_S(O)OH upon irradiation at 266 nm (22 min, 16 K). The symbol “^16/18^O” refers to a 1:1 mixture of the species containing ^16^O and ^18^O.

To unravel the photochemistry of HOSO• in CO‑ and CO_2_‑matrixes, the IR spectra for the 266 nm laser photolysis products were recorded and the resulting difference spectra reflecting the reactions of HOSO• are depicted in Fig. 4. In contrast to the photodissociation of HOSO• to H•/SO_2_ and the caged complex •OH···SO in Ar‑matrix (Fig. 4a), its photolysis in solid CO (Fig. 4b) yields CO_2_, OCS, HCO•, HOCO•, H_2_CO, HOS•, and SO_2_. It is noteworthy that the IR frequencies for all these species shift slightly in comparison to those observed for the corresponding species in CO‑matrixes, indicating weak interactions between the neighboring counterpart species formed after the bimolecular reaction of HOSO• with CO inside the rigid CO‑matrix cages. For instance, the two characteristic IR bands of *t*‑HOCO• for the *ν*(O–H) and *ν*(C=O) modes at 3456 and 1833 cm^−1^ in CO‑matrix shift to 3311.0 and 1831.7 cm^−1^ due to complexation with SO (Supplementary Table 1). Concomitantly, the IR band of the counterpart SO at 1139.5 cm^−1^ (CO‑matrix) undergoes blue‑shift to 1140.0 cm^−1^, and the deformation mode *δ*(COH) exhibits a larger blue‑shift of +12.8 cm^−1^. The presence of the less stable conformer *c*‑HOCO• is evidenced by the band at 1795.2 cm^−1^, and it is also slightly red‑shifted comparing to the band at 1797 cm^−1^ for *c*‑HOCO• in CO‑matrix^18^. Conformational conversion of *c*‑HOCO• to the lower‑energy *t*‑HOCO• happens upon subsequent irradiation at 532 nm. However, the previously reported^45^ spontaneous transformation of *c*-HOCO• → *t*‑HOCO• in N_2_‑matrix (4.5 K) via quantum mechanical tunneling was not observed in CO-matrix (16 K), which is consistent with the frequently observed environmental effects on the tunneling processes in low‑temperature matrixes^48^.

Consistent with the photodecomposition of HOSO• (→ H• + SO_2_) in Ar‑matrix, its photolysis in solid CO also causes H–O bond fragmentation followed by CO‑trapping of the mobile hydrogen atoms to afford HCO•, which acts as a hydrogen donor through weak interaction with the counterpart SO_2_ in the same CO-matrix cage by forming complex HCO•···SO_2_. In this complex, the *ν*(C‑H) mode shifts to 2493.8 cm^−1^ in comparison to the same mode at 2488 and 2483 cm^−1^ for HCO• in CO‑ and Ar‑matrixes^49^. The *ν*(C = O) and *δ*(COH) modes in HCO• and the two stretching modes of SO_2_ display small red-shifts (Supplementary Table 2) in the complex. The changes of the two stretching modes of SO_2_ in HCO•···SO_2_ (Δ*ν*_asym_ = –8.2 cm^−1^; Δ*ν*_sym_ = –0.3 cm^−1^) are smaller than those observed in other SO_2_‑contaning complexes such as H_2_O_2_···SO_2_ (Δ*ν*_asym_ = –12.9 cm^−1^; Δ*ν*_sym_ = –2.1 cm^−1^)^50^. Further combination of HCO• with mobile hydrogen atoms in the matrix during the laser photolysis affords H_2_CO, and it is also perturbed by the neighboring molecule (SO_2_) as evidenced by the appearance of two IR bands at 1736.7 and 1734.9 cm^−1^ for the *ν*(C = O) mode in H_2_CO. Note, formation of HCO• and H_2_CO has been previously observed in a solid CO/H_2_ ice mixture at 8 K after irradiation with ultra‑high vacuum UV (~160 nm) light^19^.

**Fig. 5:**
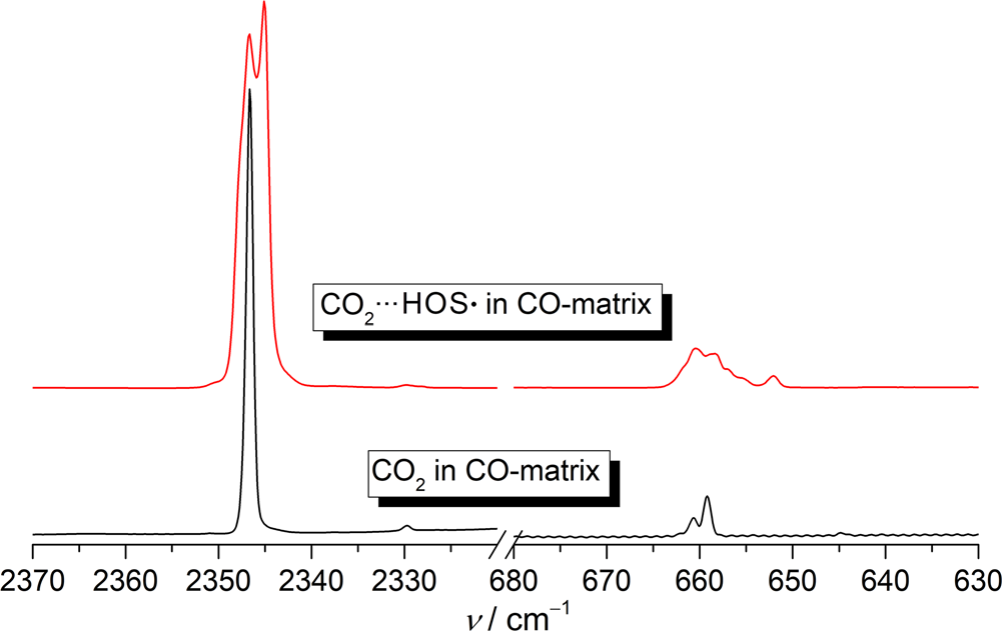
Sections of Infrared (IR) spectra showing the two bands for the two vibrational modes of CO_2_ in CO‑matrix isolated CO_2_ and CO_2_···HOS•. The lower trace corresponds to the IR spectrum of CO_2_ in CO-matrix at 16 K. The upper trace corresponds to the IR spectrum of CO_2_···HOS• in CO-matrix at 16 K.

The mechanism for the formation of HOS• during the photolysis of HOSO• in CO-matrix is intriguing. A plausible pathway is the HAT in HOCO•···SO ( → CO_2_···HOS•). In the CO_2_···HOS• complex, the *ν*(O–H) mode shifts by –154.3 cm^−1^ in comparison with the same mode observed in HOS• in solid *para*‑H_2_^51^, while the *ν*(S–O) mode displays a blue-shift of +8.9 cm^−1^ (Supplementary Table 3). Accordingly, the two IR bands for CO_2_ at 2346.7 and 659.3 cm^−1^ exhibit shoulders that can be assigned to the complex (Fig. 5). Particularly, the bending mode *δ*(CO_2_) in the complex appears as a broad band in the range of 665–650 cm^−1^ due to removal of vibrational degeneracy upon complexation with HOS•. Similar splitting for the nondegenerate *δ*(CO_2_) modes has been observed in other CO_2_‑complexes such as HKrCCH···CO_2_^52^.

The spectroscopic identification of the photoproducts of HOSO• in CO‑matrix is further supported by ^18^O‑isotope labeling experiments. Using ^18^O‑labeled sulfinic acid as the precursor, a 1:1:1:1 mixture of HOSO•, H^18^OSO•, HOS^18^O•, and H^18^OS^18^O• can be generated, and their distinction can be assured with the revolved IR bands at 1049.7, 1045.2, 1043.4, and 1039.5 cm^−1^ for the *δ*(SOH) mode in the four isotopologues, however, the remaining IR fundamental modes appear as doublets due to closeness of the isotopic shifts (Fig. 4d). The photochemistry of these isotopologues in CO-matrix (Fig. 4e) provides useful information for probing the reaction mechanism. The sole formation of HCO• (without HC^18^O•) and a 1:2:1 mixture of SO_2_, OS^18^O, and S^18^O_2_ among the photoproducts confirms the route for the straightforward HAT from HOSO• to CO. The absence of the IR bands for HOC^18^O• and H^18^OC^18^O• rules out the possibility for the formation of HOCO• via direct hydrogenation of CO_2_, since the singly ^18^O‑enriched CO_2_ is present in the same matrix. In contrast, the association of the photolytically generated •OH/•^18^OH with CO yields the experimentally observed HOCO• and H^18^OCO• in 1:1 ratio.

In the IR difference spectrum for the ^18^O-labeling experiments (Fig. 4e), each band for the *ν*(O–H) and *ν*(S–O) modes in HOS• splits into doublet due to the additional presence of H^18^OS•, the corresponding ^16/18^O-isotopic shifts –10.8 and –31.5 cm^−1^ show good agreement with the calculated values –8 and –31 cm^−1^, respectively. Assuming a bimolecular reaction of HOCO• and SO for the formation of HOS• and CO_2_, the observation of more CO_2_ than HOS• (based on the experimental and calculated IR band intensities) in the photochemistry indicates that there is an alternative pathway for producing CO_2_. Given the generation of HO• and SO in the photolysis of HOSO•, the photochemistry of SO in CO ice was also studied. Co-condensation of gaseous SO in the presence of CO (ca. 1:1000) at 16 K also yields OSSO, which can be completely destroyed by UV‑light irradiation at 365 nm (Supplementary Fig. 3). The formation of SO_2_ and OCS coincides with the photodissociation of OSSO to SO_2_ and sulfur atom with subsequent CO-trapping reaction (S + CO → OCS). When changing the irradiation source to a 193 nm laser, depletion of SO occurs by forming CO_2_ and OCS (Supplementary Fig. 3), and the mechanism can be reasonably explained that monomeric SO dissociates to sulfur and oxygen atoms followed by association reactions with CO. Therefore, formation of CO_2_ and OCS in the photochemistry of HOSO• in solid CO is very likely caused by the photofragmentation of the initially generated SO. This mechanism is also consistent with the sole observation of none-isotopically labeled OCS and 1:1 mixture of CO_2_/OC^18^O in the ^18^O‑lalebing experiments (Fig. 4e). Additionally, traces of HCO• form during laser irradiation of the impurity H_2_O in solid CO.

The photolytic depletion of HOSO• in CO‑matrix at 266 nm is extremely fast, and the depletion becomes much less efficient under subsequent UV‑light irradiation at 365 nm (Supplementary Fig. 4 and 5). Prolonged 266 nm laser irradiation (36 min) leads to complete depletion of HOSO•. Further irradiation at 365 nm promotes reverse HAT from HOCO• to SO_2_ by reforming HOSO• and CO_2_. The photosensitivity of HOCO• towards the UV‑light (365 nm) strongly indicates that the aforementioned weak absorptions in the UV‑vis spectra in the range of 300 − 400 nm (Fig. 3) belongs to this carbonyl radical. It is also consistent with the observation in the IR spectra that HOCO• forms after the photolysis of HOSO• in CO‑matrix (Fig. 4). The absorption for HCO• above 400 nm^53^ was not observed in the UV‑vis spectra due to low intensity.

The photo‑induced HAT of HOSO• also occurs in CO_2_-doped Ar-matrix at 16 K (Fig. 4c), yielding a new molecular complex HOCO•···SO_2_ (Supplementary Table 1). The strongest band for the *ν*(C = O) mode in the complex locates at 1818.9 cm^−1^, and it is lower than the same mode in HOCO•···SO and HOCO• at 1831.7 and 1833 cm^−1^, respectively. The very weak *δ*(COH) mode undergoes small blue‑shift to 1278.7 cm^−1^ comparing to the frequencies of 1261 and 1265.8 cm^−1^ for HOCO• in solid CO‑matrix and in HOCO•···SO. In contrast, the two SO_2_ stretching modes at 1350.9 and 1146.4 cm^−1^ are red-shifted in comparison to free SO_2_ at 1355.0 and 1152.2 cm^−1^. Unlike the generation of two conformers of HOCO• in the photochemistry of HOSO• in CO‑matrix (Fig. 4b), only one conformer was generated in the photolysis of HOSO• in CO_2_‑doped Ar‑matrix (Supplementary Fig. 2). Additional formation of OCS among the photolysis products implies the trapping reaction of sulfur atoms (SO → S + O) by CO_2_, however, the elusive intermediate OSCO^54^ was not observed. Formation of OCS and HCO• was also observed during the 193 nm laser irradiation of a mixture of H_2_O and SO_2_ in CO‑matrix (Supplementary Fig. 6).

### Calculated structures of molecule-radical complexes

**Fig. 6:**
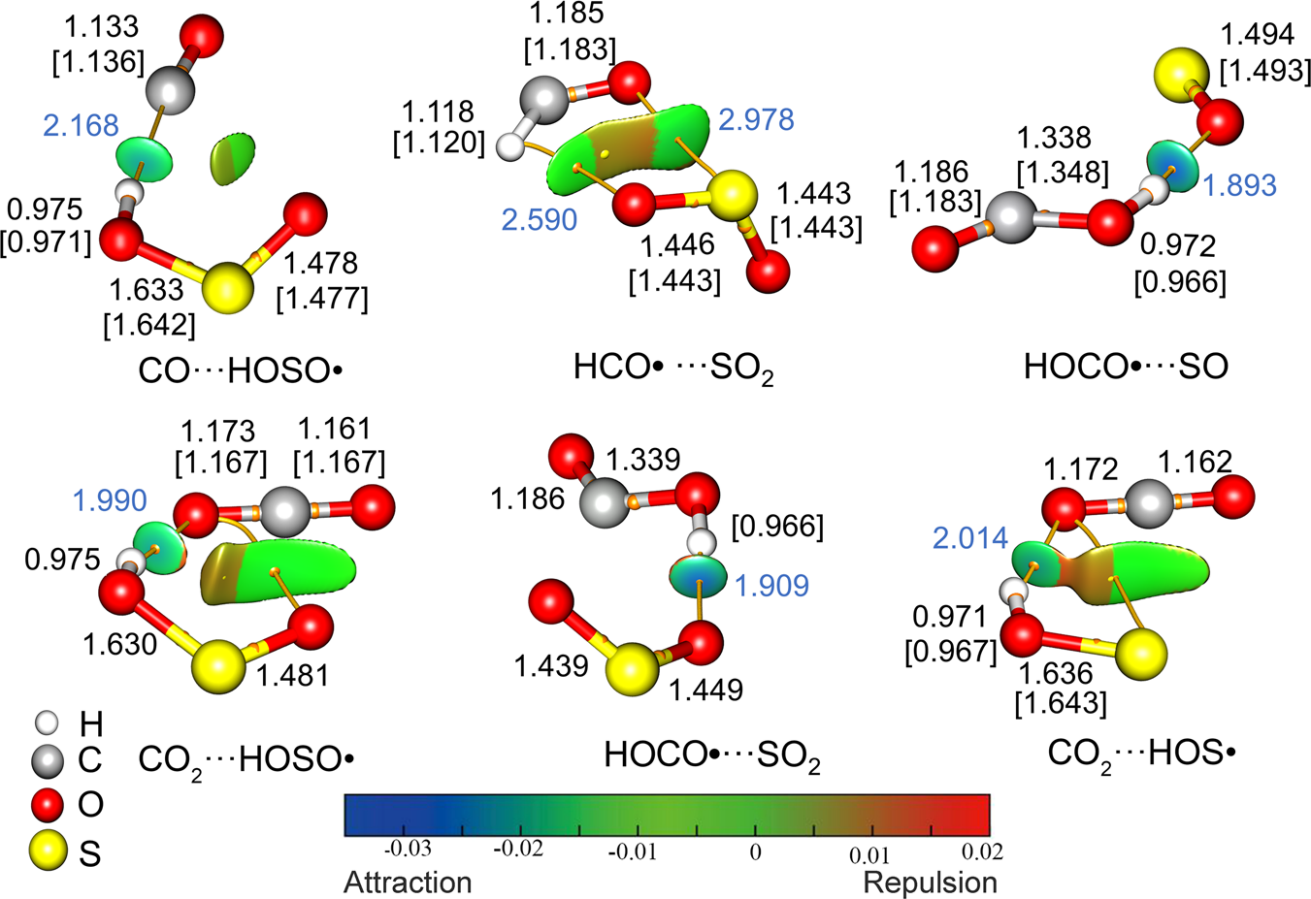
The CCSD(T)/aug-cc-pV(T + D)Z calculated molecular structures and noncovalent interaction analysis for all the observed complexes. The bond lengths (Å) for the monomers are given in square brackets, and the lengths for the intermolecular hydrogen bonds are shown in blue. The calculated gradient isosurfaces (*s* = 0.6 au) are colored on a blue‑green‑red scale according to values of sign(*λ*_2_)*ρ*, ranging from –0.035 to 0.02 au. Blue indicates strong attractive interactions, and red indicates strong repulsive interactions.

Weakly bonded complexes consisting of simple radicals (e.g., HO• and HOO•) and small molecules (e.g., CO, CO_2_, and H_2_O) play important roles in gas‑phase chemistry, as they may serve as key intermediates in clusters formation in atmospheric and astrochemical processes at the low‑temperature surface of dust and ice grains^39^. Therefore, the structures and reactivity of these molecule‑radical complexes have been the focus of comprehensive experimental and computational studies. Despite the importance of HOSO•, HOCO•, and HCO• in gas-phase chemistry has been increasingly recognized, their complexes remain scarcely investigated. Using the UCCSD(T)/aug‑cc‑pV(T + D)Z method, the structures, energies, and bonding properties for the new molecule-radical complexes involved in the photochemistry of HOSO• in CO and CO_2_ ices were calculated (Fig. 6).

In sharp contrast to a favorable *cis*‑planar structure for free HOSO•, the HOSO moiety in CO···HOSO• and CO_2_···HOSO• are nonplanar. The global minimum of CO···HOSO• prefers OH···CO linkage and the hydrogen atom is dramatically tilted out of the OSO plane by 33.9°. The corresponding hydrogen bond length is 2.168 Å, which is shorter than the hydrogen bond in •OH···CO (2.341 Å)^55^. The binding energy (*D*_e_ = 3.9 kcal mol^−1^) is higher than those in •OH···CO (*D*_e_ = 2.3 kcal mol^−1^)^56^ and •OH···OS (*D*_e_ = 3.2 kcal mol^−1^)^57^. The isomer bearing a OH···OC linkage between HOSO• and CO is less stable by ca. 5 kcal mol^−1^. The hydrogen atom in CO_2_···HOSO• is also tilted from the OSO plane by a dihedral angle of 30.5°, and the CO_2_ molecule is slightly bent by 1.8°. The shorter hydrogen bond in CO_2_···HOSO• (1.990 Å) than that in CO···HOSO• (2.168 Å) coincides with the larger stabilizing interaction (*D*_e_ = 5.3 kcal mol^−1^). The noncovalent interaction (NCI) analysis suggests strong attractive hydrogen bond interactions and simultaneous weak repulsive interactions of the terminal S = O moiety with CO and CO_2_ in these complexes (Fig. 6).

Unlike the distortion of the complexed *cis*‑HOSO•, the HOCO moiety in the hydrogen-bonded HOCO•···SO and HOCO•···SO_2_ complexes keeps a favorable *trans*‑planar configuration. The shorter hydrogen bond in the HOCO•···SO (1.893 Å) than that in the latter (1.909 Å) is also consistent with a higher stabilizing energy of 6.1 kcal mol^−1^ (5.4 kcal mol^−1^ in HOCO•···CO_2_). The binding energy in HOCO•···SO is larger than that in the hydrogen-bonded H_2_O···SO complex (*D*_e_ = 3.1 kcal mol^−1^)^58^, while the latter remains yet experimentally unobserved. The weak interaction between HCO• and SO_2_ in HCO•···SO_2_ (*D*_e_ = 3.5 kcal mol^−1^) is facilitated by forming a five‑membered ring through concerted contacts of hydrogen bond CH–OS (2.590 Å) and chalcogen bond CO–SO (2.978 Å). Similar five‑membered ring structure has also been predicted for the detectable HO_2_•···SO_2_ complex (*D*_e_ = 4.6 kcal mol^−1^)^59^. The hydrogen bond interaction in the planar CO_2_···HOS• complex (*D*_e_ = 4.5 kcal mol^−1^) is stronger than the intermolecular O···C contact in the T‑shaped van der Waals complex H_2_O···CO_2_ (*D*_e_ = 2.8 kcal mol^−1^)^60^.

Energetically, HCO•···SO_2_ and HOCO•···SO are higher than CO···HOSO• by 37.5 and 27.1 kcal mol^−1^, respectively, whereas the secondary HAT in HOCO•···SO to form CO_2_···HOS• is highly exothermic by releasing –51.5 kcal mol^−1^. Therefore, the overall process for the oxidation of CO to CO_2_ by reaction with HOSO• is exothermic by –14.0 kcal mol^−1^, which is comparable with the energy (−20.0 kcal mol^−1^) for the •OH radical promoted oxidation^14^. The HAT process in CO_2_···HOSO• to form HOCO•···SO_2_ is endothermic by 38.5 kcal mol^−1^, which is lower than the H–O bond dissociation energy in HOSO• (44.1 kcal mol^−1^)^57^.

### Implications in interstellar sulfur chemistry

Our experimental results demonstrate that organic radicals HCO• and HOCO• can be produced by UV-irradiation of HOSO• in astronomical CO and CO_2_ ices via the hydrogen atom transfer (HAT) reactions of the initially formed molecular complexes at 16 K, and the acyl radicals also form stable molecular clusters with sulfur oxides through strong hydrogen bonding interactions. Considering the facile formation of HOSO• from the photoreactions of SO_2_ with H_2_O, the generation of the two important building blocks HCO• and HOCO• from the photoreactions mimics the chemical evolution network of the dehydrogenated sulfur-containing molecules SO and SO_2_ in the outer layer of the CO-dominant interstellar icy grains in molecular clouds at a typical temperature of about 10 K. Alternatively, other radicals (e.g., •CH_3_, •CH_2_OH, and •CN) derived from the photoreactions of the interstellar carbon- or nitrogen-containing molecules (e.g., CH_4_, CH_3_OH, and HCN) in the cryogenic astronomical ices may also be present and react further with the acyl radicals through barrierless radical-radical association reactions to form more complex organic molecules (COMs). Additionally, the formation of OCS in the photochemistry of HOSO•, SO and SO_2_ in CO and CO_2_ ices may also contribute to understanding the interstellar sulfur chemistry, since OCS not only serves as a prebiotic activating agent for amino acid polymerization in forming peptides under mild conditions in aqueous solution^61–63^, also it involves in the reduction of CO_2_^64^ and acts as a condensing agent in phosphate chemistry^65^.

Clearly, the HAT processes of HOSO• may serve as the link connecting the chemistry of SO_2_ and the chemistry of carbon oxides (CO and CO_2_) in interstellar ices, although the ubiquity of HAT in chemistry, biology, and industry has been well recognized^66^. The uncovered chemical network for the formation of the astrochemically relevant organic radicals HCO• and HOCO• in the simple systems containing the primordial substrates (e.g., H_2_O, SO, SO_2_, CO, and CO_2_) might aid in disclosing the intriguing mechanism for the chemical evolution of biomolecules such as organic acids in dense molecular clouds, where barrierless radical-radical reactions at low temperatures (10–20 K) are assumed to happen spontaneously for the formation of COMs^67,68^. Furthermore, the spectroscopic identification and photochemistry of the new complexes consisting of the astrochemically relevant radicals HOSO•, HCO•, HOCO•, and HOS• will help in understanding the chemical composition and abundances in the interstellar medium, since molecular complexes are known to contribute to the formation of interstellar media and nucleation of aerosols in diverse planetary atmospheres, such as the CO‑rich interstellar comet 2I/Borisov^69^ and SO_2_‑rich atmosphere of Venus^70^.

Aside from the role in ice-grain photochemistry, the planetary atmospheric chemistry of sulfur-containing species also attracts enormous interest due to importance in astrochemical reactions and planetary geology. Particularly, the photochemistry of SO_2_ plays a fundamental role in the sulfur cycle in the Venusian atmosphere. In addition to the primary contribution from the volcanic eruptions^71^, photolysis of H_2_SO_4_ vapor is another source of SO_2_ as the planet is completely enshrouded by the acid droplets clouds. As demonstrated by previous field measurements^72^ and modeling^34^, photolysis of H_2_SO_4_ vapor yields SO_3_ and H_2_O, followed by further photodecomposition of SO_3_ to SO_2_ and SO. This simple photochemistry can enhance the abundances of SO_2_ (66 ± 5 ppb) and SO (31 ± 4 ppb) in the cold (ca. –80 °C) mesosphere of Venus at altitudes of 85–100 km^73^, where the CO abundances (ca. 30 ppm) vary with altitude partially due to reactions with sulfur compounds (e.g., SO_2_, SO, and S_2_)^74^. Hence, the disclosed photochemical reactions between sulfur oxides and carbon oxides in the presence of H_2_O (an average abundance of 30 ppm at 30–45 km altitude)^75^ may affect the composition of Venusian atmosphere. Recently, Limaye et al.^76^ proposed that the lower cloud layer of Venus (50–70 km) is an important target for study, since biorelevant organic acids might be generated through the iron-catalyzed metabolic redox reactions of the abundant CO_2_, CO, H_2_O, and SO_2_ under favorable chemical and physical conditions. The observed photoreactions of sulfur-containing species with CO_2_ and CO at low temperatures suggests that the hydrogenation of carbon oxides to the organic radicals HCO• and HOCO• for the formation of organic acids might be facilitated by HAT reactions with the derived radicals HOSO• and HSO•.

## Methods

### Sample preparation

Difluoromethylsulfinic acid (CHF_2_S(O)OH) was synthesized by reaction of hydrogen chloride (HCl) with sodium difluromethylsulfinate (CHF_2_S(O)ONa)^44^. Specifically, freshly dried HCl (10 mmol) was condensed into a glass vessel containing solid CHF_2_S(O)ONa (0.14 g, 1 mmol) at –196 °C (liquid nitrogen bath), and the mixture was warmed to −110 °C (cold ethanol bath) and kept for overnight reaction. Then, the reaction mixture was slowly warmed to ca. −60 °C and the volatile part was pumped (10 pa) through the vacuum line consisting of two successive cold U-traps at −60 (cold ethanol bath) and −196 °C, and the acid CHF_2_S(O)OH (ca. 50 mg, 0.5 mmol) was obtained in the first cold trap. The ^18^O-enriched CHF_2_S(O)OH was synthesized through hydrolysis of CHF_2_S(O)Cl with water (^18^O, 97%, Eurisotop), from which a 1:1:1:1 mixture of CHF_2_S(O)OH, CHF_2_S(^18^O)OH, CHF_2_S(O)^18^OH, and CHF_2_S(^18^O)^18^OH (Supplementary Fig. 7) was obtained according to the IR spectrum of its decomposition product HOSO• and also the IR spectrum of its reaction product (SO_2_) with CO (Fig. 4e).

### Matrix‑isolation spectroscopy

Matrix infrared (IR) spectra are recorded using an FT‑IR spectrometer (Bruker 70 V) in a reflectance mode with a transfer optic. A KBr beam splitter and liquid-nitrogen-cooled mercury cadmium telluride (MCT) detector are used in the mid‑IR region (5000‒450 cm^−1^). For each spectrum, 200 scans at a resolution of 0.5 cm^−1^ are co‑added. Matrix ultraviolet-visible (UV‒vis) spectra in the range of 190‒800 nm are recorded using a Perkin Elmer Lambda 850+ spectrometer with a scanning speed of 1 nm s^−1^.

For the preparation of the matrix, gaseous sample is mixed by passing matrix gas (Ar, CO or CO_2_) through a cold U‑trap (−10 °C) containing ca. 50 mg of the acid precursor (CHF_2_S(O)OH). Then, the mixture of acid vapor in matrix gas (a ratio of ca. 1:1000) is passed through an aluminum oxide furnace (2.0 mm, i.d.:1.0 mm), which can be heated over a length of ∼25 mm by a tantalum wire (o.d. 0.4 mm, resistance 0.4 Ω) and immediately deposited (2 mmol h^−1^) in a high vacuum (∼10^−5^ Pa) onto a gold‑plated copper block matrix support (10 K for Ar‑matrix, 16 K for CO‑matrix) for IR spectroscopy and onto a CaF_2_ window (16 K) for UV‒vis spectroscopy using closed‑cycle helium cryostats (Sumitomo Heavy Industries, SRDK‑408D2‑F50H) inside the vacuum chambers. For the preparation of the CO_2_- or CO-doped Ar-matrix, a premix of Ar with CO_2_ or CO (a ratio of 1:20) was used as the matrix gas. Temperatures at the second stage of the cold heads are controlled and monitored using East Changing TC 290 (IR spectroscopy) and Lake‑Shore 335 (UV‒vis spectroscopy) digital cryogenic temperature controller silicon diodes (DT‑670). The voltage and current use in the pyrolysis experiments are 7 V and 2.9 A, respectively. Photolysis experiments were performed using the Nd^3+^:YAG laser (266 nm, MPL‑F‑266, 10 mW) and UV lamp (365 nm, 24 W).

### Quantum chemistry calculation

Molecular geometries of stationary points for the monomers and complexes were first calculated at the B3LYP‑GD3(BJ)/def2-TZVP^77^ level of theory with the Gaussian 16 software package^78^. The dispersion correction using the D3 version of Grimme’s dispersion with Becke-Johnson damping, GD3(BJ)^79^, is necessary to obtain reliable structures for the hydrogen-bonded complexes. Then, the structures were further optimized at the CCSD(T)/aug‑cc‑pV(T + D)Z^80^ level of theory with MOLPRO (ver. 2019.1) software^81^. The non‑covalent interactions (NCIs) analyses are carried out at the B3LYP‑GD3(BJ)/def2‑TZVP level of theory with Bader’s quantum atoms‑in‑molecules (QTAIM)^82^ and Johnson’s NCI analyses^83^ on basis of the CCSD(T)/aug‑cc‑pV(T + D)Z level of theory optimized structures.

## Supplementary information

Supplementary Information

Peer Review File

## Data Availability

All data to evaluate the conclusion in the paper are available in the main text and/or the Supplementary Materials. The atomic coordinates generated in this paper have been deposited in the ZENODO database. (https://zenodo.org/record/7262494#.Y1zQn3ZBy3B).
