# Supplementary material for: Unraveling sulfur chemistry in interstellar carbon oxide ices
Source: Nat Commun. 2022 Nov 22;13:7150. doi: 10.1038/s41467-022-34949-4 (PMC9684543; doi:10.1038/s41467-022-34949-4)
Supplement: Supplementary file 1 — Supplementary Information [file 41467_2022_34949_MOESM1_ESM.pdf]

Supplementary information

Unraveling Sulfur Chemistry in Interstellar Carbon Oxide Ices

Xiaolong Li, Bo Lu, Lina Wang, Junfei Xue, Bifeng Zhu, Tarek Trabelsi, Joseph S. Francisco, and Xiaoqing Zeng

---

In the format provided by the authors and unedited

Supplementary information file includes:

IR spectra obtained in matrixes (Figs. 1–6)..2

Pathway for the synthesis of ^{18}O-labeled HOSO• (Fig. 7)..6

Calculated and observed IR spectra of HOCO• and its complexes (Table 1)..................................7

Calculated and observed IR spectra of HCO• and its complexes (Table 2)......................................8

Calculated and observed IR spectra of HOS• and its complexes (Table 3)......................................8

Supplementary References...8

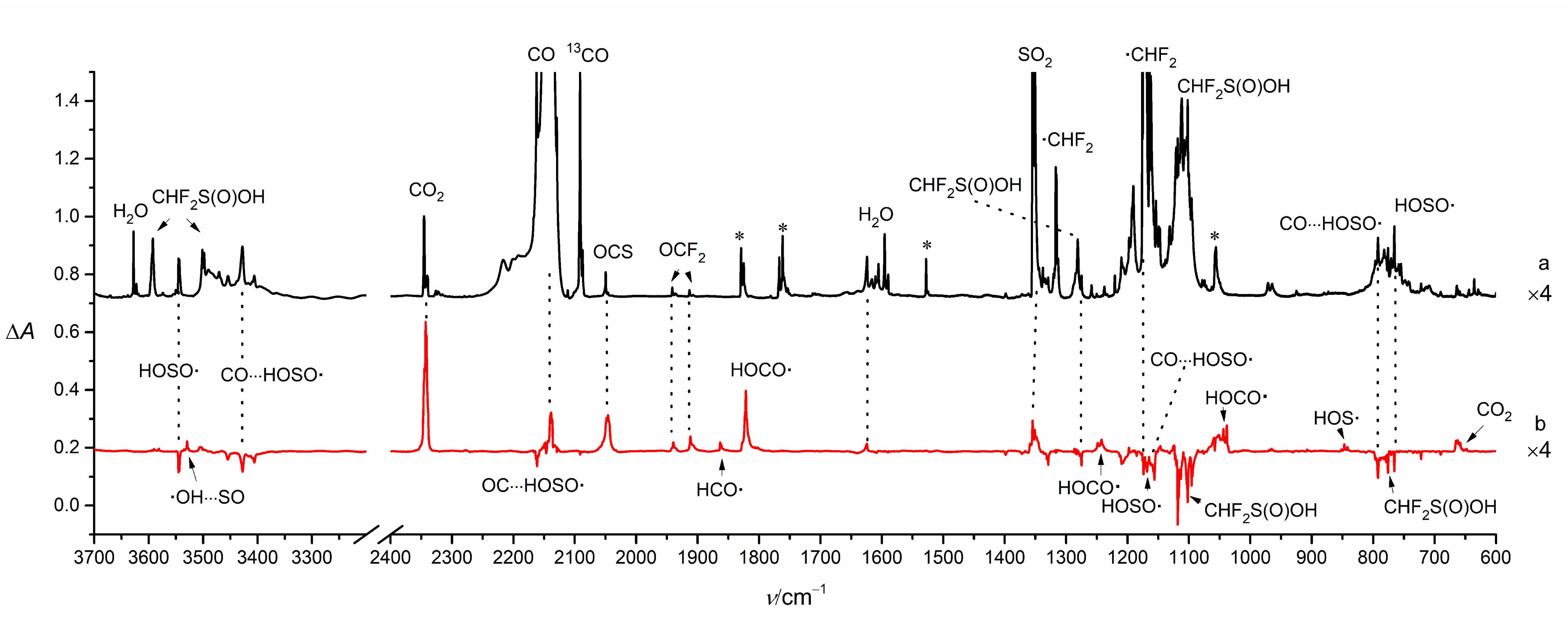

Supplementary Fig. 1 | The radical complexes and photochemistry of HOSO• in CO-doped Ar-matrix. a Infrared (IR) spectrum (3700–600 cm^{-1}) for the high-vacuum flash pyrolysis (HVFP, ca. 700 °C) products of $CHF_2S(O)OH$ in CO-doped Ar-matrix (1:50) at 16 K. **b** IR difference spectrum (3700–600 cm^{-1}) reflecting the changes of the HFVP products of $CHF_2S(O)OH$ in CO-doped Ar-matrix (20 : 1000) at 16 K upon 30 min 266 nm laser irradiation. Unknown species (*) are marked.

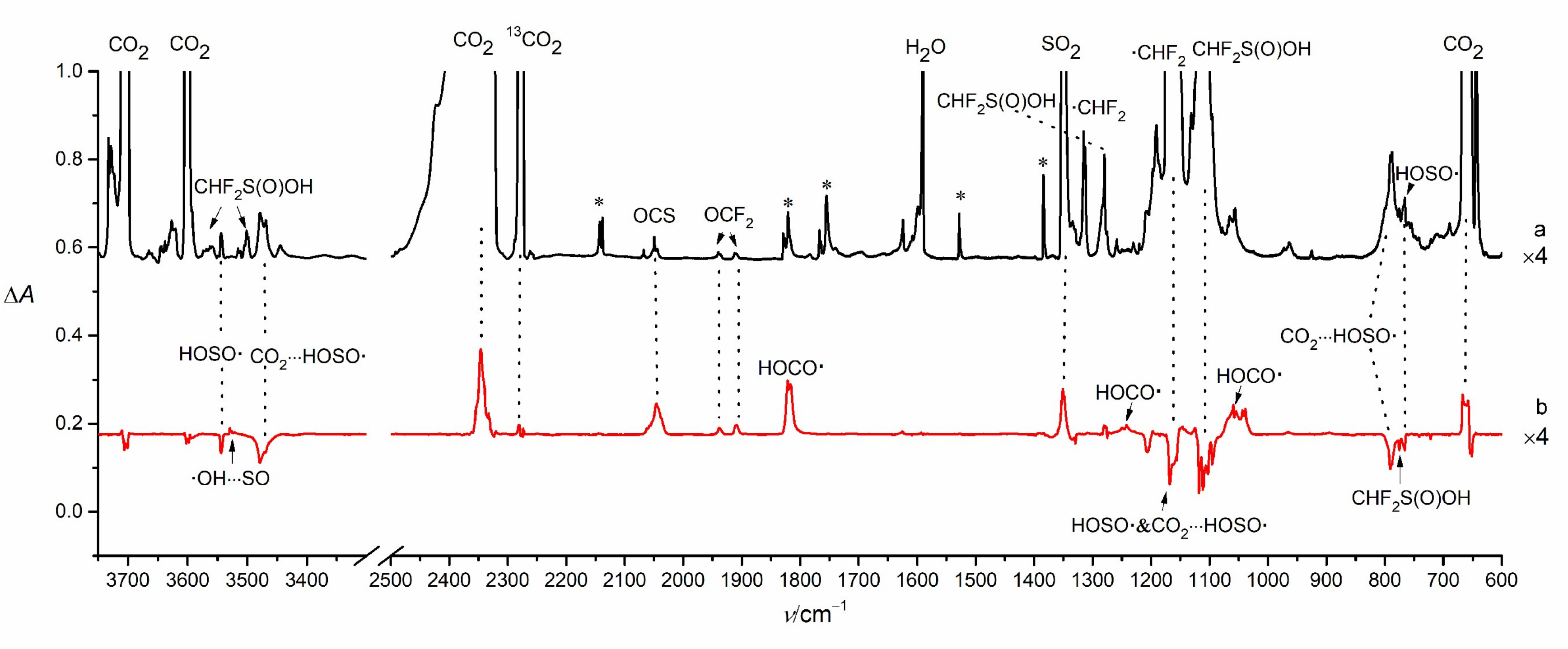

Supplementary Fig. 2 | The radical complexes and photochemistry of HOSO in CO_2-doped Ar-matrix. a Infrared (IR) spectrum (3750–600 cm^{-1}) for the HVFP products of $CHF_2S(O)OH$ in CO_2-doped Ar-matrix (50:1000) at 16 K. **b** IR difference spectrum (3750–600 cm^{-1}) reflecting the changes of the HFVP products of $CHF_2S(O)OH$ in CO_2-doped Ar-matrix (20 : 100) at 16 K upon 30 min 266 nm laser irradiation. Unknown species (*) are also marked.

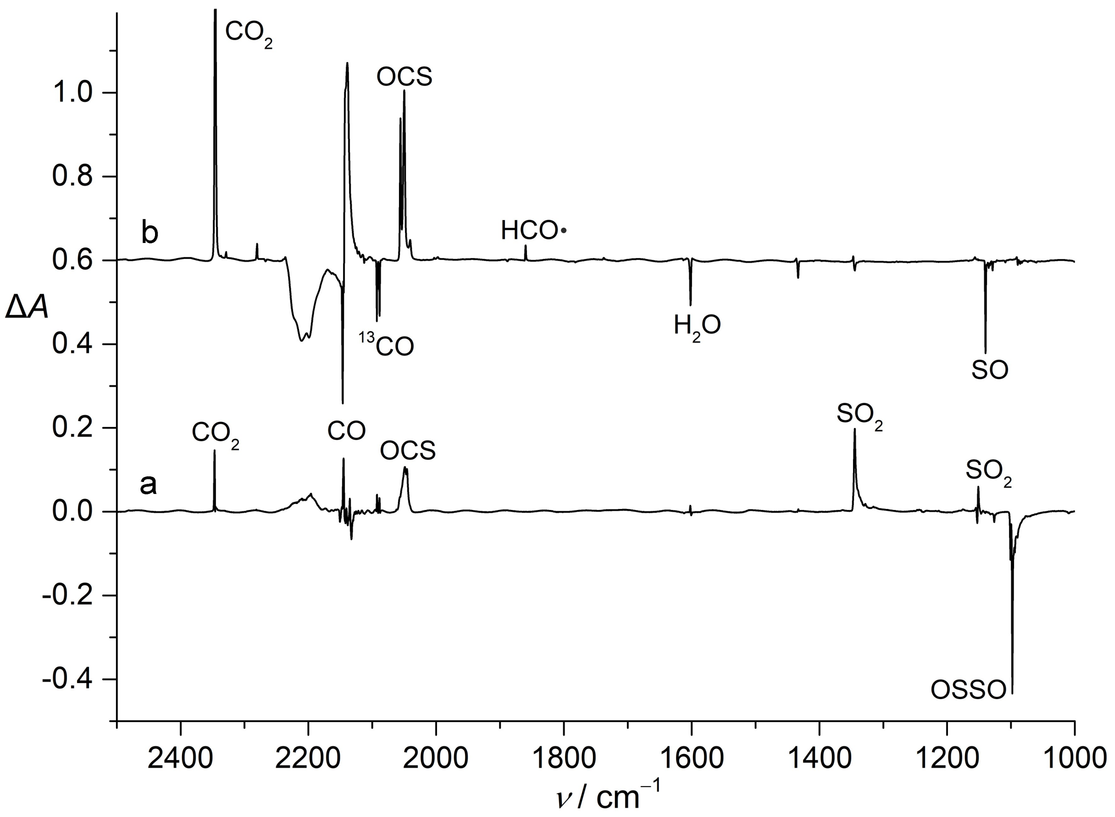

Supplementary Fig. 3| Reactions of OSSO and SO in matrixes upon irradiation. a Infrared (IR) difference spectrum reflecting the change of the CO-matrix isolated OSSO upon irradiation at 365 nm (15 min). **b** IR difference spectrum reflecting the change of the CO-matrix isolated SO upon irradiation at 193 nm (3 min).

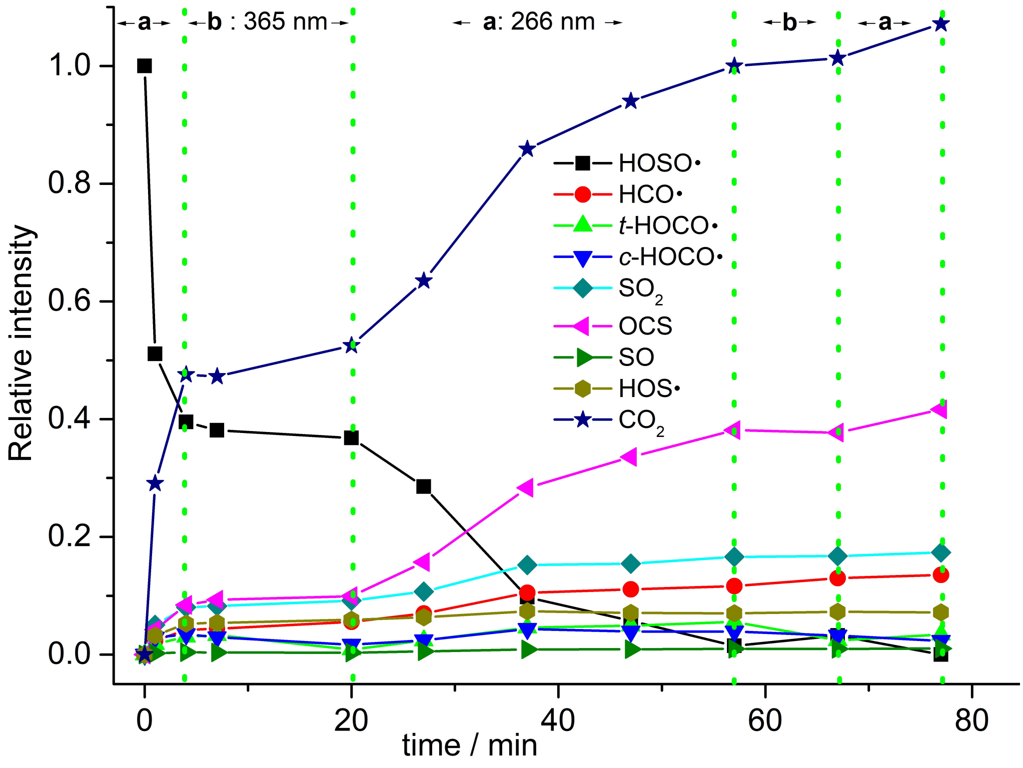

Supplementary Fig. 4 | The integrated infrared band intensities during the irradiation of HOSO• in CO-matrix. For clarity, the initial intensity of HOSO• was normalized. Panel **a** corresponds to the irradiation the matrix at 266 nm, and panel **b** corresponds to the irradiation of the matrix at 365 nm.

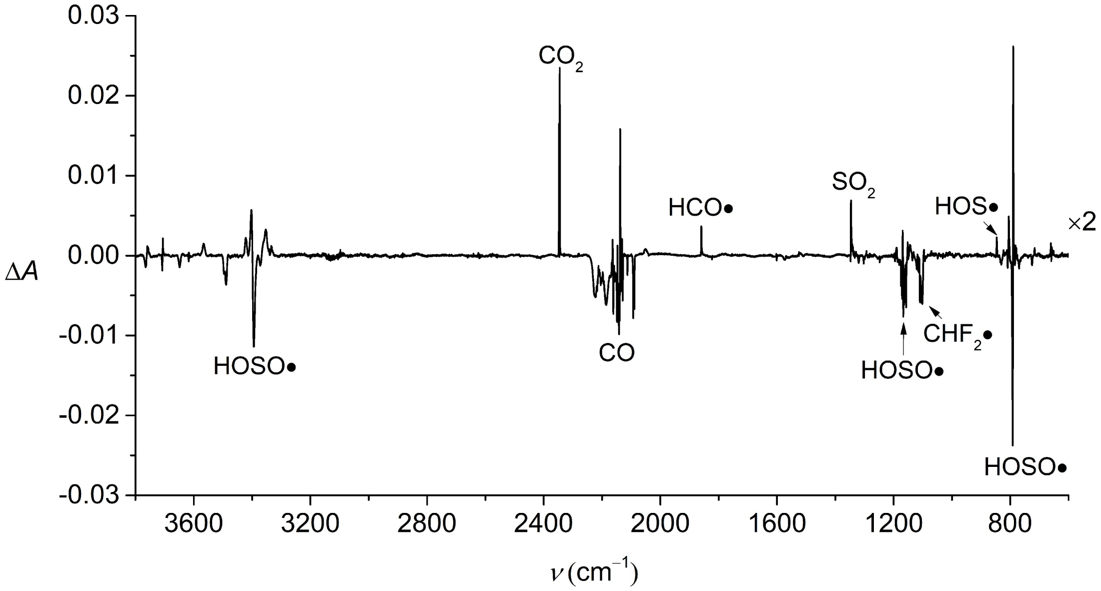

Supplementary Fig. 5 | The photochemistry of HOSO• in CO-matrix. Infrared (IR) difference spectrum reflecting the change of the CO-matrix isolated HOSO• upon irradiation at 365 nm (7 min).

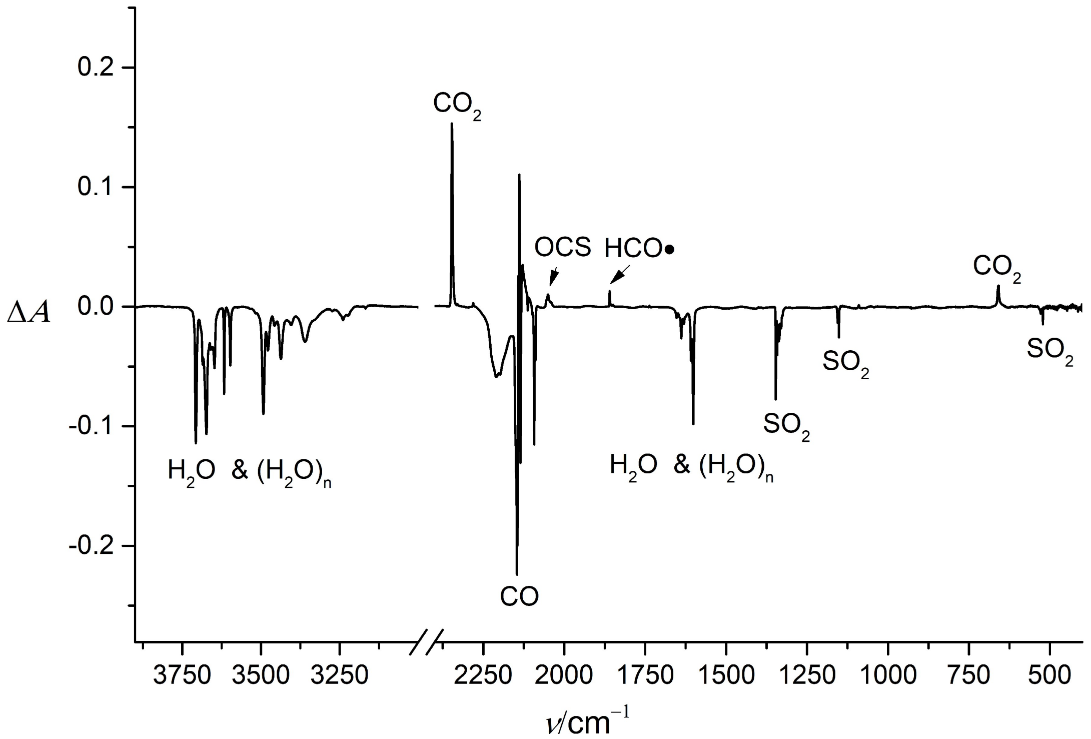

Supplementary Fig. 6 | The photochemistry of a $SO_2/H_2O/CO$ (1 : 10 : 1000) mixture. Infrared (IR) difference spectrum reflecting the change of the CO-matrix isolated H_2O and SO_2 upon irradiation at 193 nm (14 min).

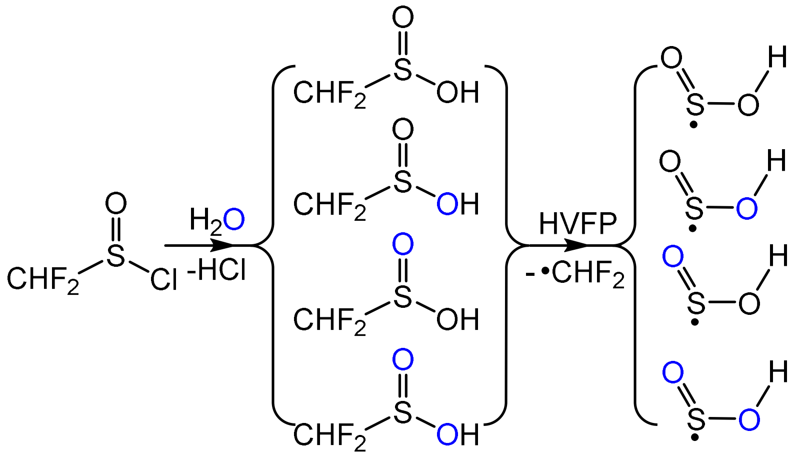

Supplementary Fig. 7| Pathway for the synthesis of ^{18}O-labeled HOSO• by using ^{18}O-labeled H_2O (97%) as the starting material.

Supplementary Table 1 | Calculated and observed Infrared (IR) spectra of HOCO•/SO/SO_2 and their complexes.

Mode	*t*-HOCO•/SO/SO_2 [a]			*t*-HOCO•···SO			*t*-HOCO•···SO_2		
	Obs.[a]	B3LYP[b]	CCSD(T) [c]	Obs.[d]	B3LYP[b]	CCSD(T) [c]	Obs.[e]	B3LYP[b]	CCSD(T) [c]
ν(O–H)	3456	3779 (119)	3804	3311.0	3607 (633)	3861	3529.1	3615 (597)	3686
ν(C=O)	1833	1902 (230)	1882	1831.7/1795.2	1888 (271)	1870	1818.9	1890 (274)	1871
δ(COH)	1261	1235 (244)	1254	1273.8	1306 (190)	1319	1278.7	1313 (193)	1358
$\nu_{asym}(SO_2)$	1355.0	1376 (215)	1365	-	-		1350.9	1368 (210)	13221
$\nu_{sym}(SO_2)$	1152.2	1179 (32)	1156	-	-		1146.4	1176 (53)	1157
ν(SO)	1139.5	1157 (15)	1144	1140.0	1164 (45)	1149	-	-	-

[a] Observed IR frequencies in CO-matrix.[1] [b] Calculated harmonic IR frequencies and intensities (km mol^{-1}, in parentheses) at the B3LYP-D3(BJ)/def2-TZVP level of theory. [c] Calculated harmonic IR frequencies at the CCSD(T)/aug-cc-pV(T+d)Z level of theory. [d] Observed IR frequencies in CO-matrix. [e] Observed IR frequencies in CO_2-doped Ar-matrix.

Supplementary Table 2 | Calculated and observed Infrared (IR) spectra of HCO•/SO_2 and their complex.

Mode	HCO•/SO_2			HCO•···SO_2		
	Obs.[a]	B3LYP[b]	CCSD(T) [c]	Obs.[d]	B3LYP[b]	CCSD(T) [c]
ν(C–H)	2488	2645 (81)	2703	2493.8	2727 (18)	2750
ν(C=O)	1861	1935 (96)	1876	1859.9	1922 (87)	1868
δ(COH)	1090	1108 (36)	1108	1076.5	1101 (101)	1120
$\nu_{asym}(SO_2)$	1355.0	1376 (215)	1365	1346.8	1358 (204)	1358
$\nu_{sym}(SO_2)$	1152.2	1179 (32)	1156	1151.9	1160 (50)	1156

[a] Observed IR frequencies in CO-matrix.[2] [b] Calculated harmonic IR frequencies and intensities (km mol^{-1}, in parentheses) at the B3LYP-GD3(BJ)/def2-TZVP level of theory. [c] Calculated harmonic IR frequencies at the CCSD(T)/aug-cc-pV(T+d)Z level of theory. [d] Observed IR frequencies in CO-matrix.

Supplementary Table 3 | Calculated and observed Infrared (IR) spectra of HOS•/CO_2 and their complex.

Mode	HOS•/CO_2			CO_2···HOS•		
	Obs.[a]	B3LYP[b]	CCSD(T) [c]	Obs.[d]	B3LYP[b]	CCSD(T) [c]
ν(O–H)	3605.7	3722 (91)	3765	3451.4	3644 (213)	3703
δ(SOH)	1156.5	1167 (46)	1185	1166.2	1224 (85)	1238
ν(S–O)	837.3	845 (68)	843	846.2	859 (55)	854
$\nu_{asym}(CO_2)$	2346.7	2411 (650)	2372	2345.1	2409 (594)	2372
$\delta(CO_2)$	659.3	677 (64)	664	652.1	671 (27)/648 (85)	660/650

[a] Observed IR frequencies in *para*-H_2 matrix.[3] [b] Calculated harmonic IR frequencies and intensities (km mol^{-1}, in parentheses) at the B3LYP-GD3(BJ)/def2-TZVP level of theory. [c] Calculated harmonic IR frequencies at the CCSD(T)/aug-cc-pV(T+d)Z level of theory. [d] Observed IR frequencies in CO-matrix.

Supplementary References

1. Milligan, D. E. & Jacox, M. E. Infrared spectrum and structure of intermediates in the reaction of OH with CO. *J. Chem. Phys.* **54**, 927–942 (1971).
2. Ewing, G. E., Thompson, W. E. & Pimentel, G. C. Infrared detection of the formyl radical HCO. *J. Chem. Phys.* **32**, 927–932 (1960).
3. Góbi, S., Csonka, I. P., Bazsó, G. & Tarczay, G. Successive hydrogenation of SO and SO_2 in Solid *para*-H_2: Formation of elusive small oxoacids of sulfur. *ACS Earth Space Chem.* **5**, 1180–1195 (2021).
